# The Role of PKC and HIF-1 and the Effect of Traditional Chinese Medicinal Compounds on Cerebral Ischemia-Reperfusion Injury

**DOI:** 10.1155/2022/1835898

**Published:** 2022-02-27

**Authors:** Zheyu Fang, Yangyang Zhang, Xixi Zhao, Weifeng Jin, Li Yu

**Affiliations:** ^1^The First School of Clinical Medicine, Zhejiang Chinese Medical University, Hangzhou 310053, China; ^2^School of Basic Medical Sciences, Zhejiang Chinese Medical University, Hangzhou 310053, China; ^3^School of Pharmaceutical Sciences, Zhejiang Chinese Medical University, Hangzhou 310053, China; ^4^School of Life Sciences, Zhejiang Chinese Medical University, Hangzhou 310053, China

## Abstract

Neuronal death occurs during cerebral ischemia. However, when hemoperfusion and oxygen supply are resumed to the ischemic focus of the brain tissue, the brain tissue damage is further aggravated, resulting in cerebral ischemia-reperfusion injury (CIRI) to the patients. Protein kinase C (PKC) plays an important role in CIRI. Through the IP3/DAG/Ca^2+^ signaling pathway, it promotes the influx of calcium ions in neurons and causes calcium overload, which aggravates the damage. At the same time, when brain cells are hypoxic, hypoxia-inducible factor-1 (HIF-1) is expressed, which regulates the expression of Bcl-2 and Bax through the PI3K/Akt signaling pathway and reduces nerve cell injury. It also fights hypoxic-ischemic injury by increasing the production of vascular endothelial growth factor (VEGF) to promote blood vessel formation. The PKC and HIF-1 signaling pathways are also linked to CIRI. HIF-1 activates the PKC and ERK pathways via the upregulation of VEGF, leading to increased Cx43 phosphorylation and dysfunction and aggravating CIRI. Existing studies have shown that certain traditional Chinese medicine (TCM) compounds regulate the PKC and HIF-1 signaling pathways and alleviate CIRI. These compounds downregulate the PKC and the activity of the PKC-related signaling pathways to alleviate CIRI. They can also promote the expression of HIF-1, increase the content of VEGF in ischemic tissues to promote the generation of blood vessels, and improve microcirculation. TCM compounds can inhibit the cascade of reactions underlying disease occurrence and development by targeting multiple components using different herbal formulations to improve the structural and material changes in the brain cells, which alleviate CIRI and protect the brain tissue. This study briefly describes the role of PKC and HIF-1, their relationship in CIRI, and the effect of TCM on them.

## 1. Introduction

Stroke is a cerebrovascular disease caused by sudden rupture or blockage of blood vessels in the brain preventing blood from flowing into the brain and contributing to blood circulation disorders in the brain and brain tissue damage [1]. It is one of the three most deadly diseases in the world. Clinical cerebral apoplexy refers to the injury caused by an abnormal blood supply. Of these, 67.3∼80.5% cases are that of ischemic cerebral apoplexy (also known as ischemic cerebrovascular disease) and the rest are hemorrhagic cerebral apoplexy. Studies have shown that severe cerebral ischemia can lead to irreversible infarction of nerve cells in the ischemic center. If there is a blood circulation disorder in the brain tissue, when the circulation and oxygen supply to the ischemic lesions of the brain are restored, the brain tissue will be more seriously damaged, resulting in a secondary cerebral ischemia-reperfusion injury (CIRI) [2]. This suggests that the treatment of cerebral ischemia should not only restore blood supply but also prevent secondary injury. Therefore, the role of a series of pathological and biochemical reactions caused by CIRI leading to neuronal damage and the effective protection of the brain tissue have always been an important topic for modern cerebrovascular disease research. Cerebral ischemia promotes the release of excitatory neurotransmitters, and excessive excitation leads to a large influx of Ca^2+^ in neurons, resulting in injury and apoptosis of nerve cells. It is noteworthy that in the mechanism of ischemia-reperfusion injury, the activation of PKC plays an important role in regulating the massive increase in intracellular Ca^2+^, leading to calcium overload [3]. At the same time, the expression of HIF-1 can promote the production of various hypoxia stress proteins [3]. However, PKC signal transduction has a special relationship with the HIF-1 signaling pathway and its factors in CIRI.

Ancient Chinese medicine books describe the treatment of cerebral ischemia using traditional Chinese medicine (TCM). According to the dialectical theory of qi and blood in TCM, cerebral ischemia is mainly caused by the imbalance of qi in the viscera, leading to “blood stasis.” Therefore, activating blood circulation and alleviating blood stasis has become an important method in treating cerebral ischemia using TCM. In recent years, identifying methods for the prevention and treatment of cerebral ischemia using TCM has become a research hotspot due to the side effects of Western medicine. The combination therapy of various TCMs may provide a multitargeted intervention for the treatment of CIRI. Preliminary studies have shown that certain TCM compounds can treat CIRI with significant efficacy by regulating the PKC and HIF-1 signaling pathways.

In summary, in the context of CIRI, the role and interaction of PKC and HIF-1, as well as the effect of TCM on them, are important topics discussed in this article.

## 2. Structure and Function of PKC

**Figure 1:**
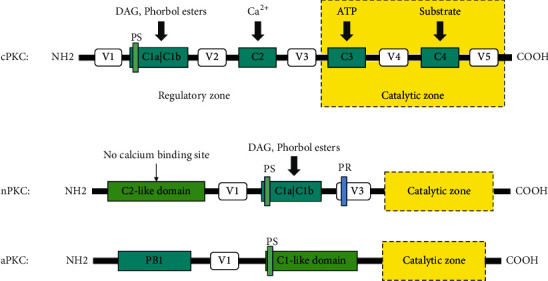
Schematic diagram of the PKC structure.

Protein kinase C (PKC), a serine/threonine protein kinase, was discovered in the cytoplasm of rat neurons in 1977 by Nishizuka, a Japanese scholar. At least 12 subtypes of PKC have been found, and different subtypes of PKC have different physical and chemical properties and immune functions [4]. PKC is ubiquitous in several vertebrates and invertebrates and plays an important role in cell signal transduction pathways. There are three types of PKC based on differences in calcium and diglycerol dependence. The structures of these types are shown in Figure 1.

① Classical conventional PKC (cPKC): due to the specific characteristics of the regulatory region, cPKC is dependent on phosphatidylserine (PS), diacylglycerol (DAG), and calcium ions. The subtypes of cPKC can be divided into *α*, *β*I, *β*II, *γ*, etc. ② Novel PKC (nPKC): nPKC is regulated only by DAG. The subtypes of nPKC include *δ*, *ε*, *θ*, and *η*. ③ Atypical PKC (aPKC): aPKC cannot be activated by calcium ions and DAG but only by PS, and its subtype include *ι*, *λ*, *ζ*, and *μ*. There is a high degree of structural homology across the different PKC subtypes, and all have the same catalytic region but different regulatory regions [5].

The conserved region (C) and the variable region (V) are two basic structural regions of cPKC. Based on the function, the cPKC structure can be divided into N-amino terminal regulatory regions (C1 and C2) and C-carboxyl terminal catalytic regions (C3 and C4) [6]. The regulatory region mainly regulates the activity of PKC. The C1 region has two “zinc finger-like regions” responsible for binding DAG or phorbol esters [7]. Acidic lipids can bind to corresponding molecular sites in the C2 region, mediating binding to calcium ions. Therefore, PS, DAG, and calcium ions determine the activity of cPKC. The V3 zone is called the hinge zone because it is responsible for connecting the regulatory and catalytic zones. The catalytic region is mainly responsible for substrate phosphorylation, the C3 region is mainly responsible for binding ATP [8], and the catalytic center is located in the C4 region.

The structure of nPKC is the same as that of cPKC, but since there is no C2 region in the regulatory region, it cannot be activated by Ca^2+^, and its activity is only regulated by DAG [6]. aPKC differs from the other two mainly in the C1 and C2 regions. The C1 region of aPKC contains only one cysteine-rich region, so aPKC lacks sensitivity to DAG. At the same time, since aPKC does not contain a C2 region, its activity is regulated not only by Ca^2+^ but also by PS [6].

PKC, also known as the third messenger of cells, is involved in several pathophysiological processes in the central nervous system [7]. PKC functions mainly through the phosphorylation of intracellular proteins, which leads to a series of physiological reactions, including gene expression, cell degeneration and proliferation, synaptic plasticity, neurotransmitter release, and programmed cell death.

## 3. Relationship between PKC and CIRI

The mechanism underlying neuronal damage caused by PKC during CIRI is mainly the theory of translocation and activation of PKC [8]. In the resting state, PKC is distributed in the cytoplasm. When stimulated by upstream signaling molecules, PKC enters the nucleus, membrane, and organelle membranes, is activated, and participates in physiological and pathological processes.

### 3.1. PKC Signaling Pathway

**Figure 2:**
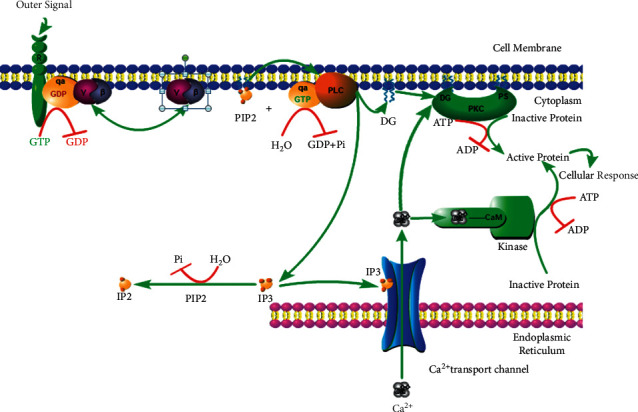
PKC signaling pathway.

The translocation and activation of PKC can promote the aggregation of intracellular Ca^2+^ to cause vasoconstriction in the brain tissue, which aggravates hypoxia and ischemia. The IP3/DAG/Ca^2+^ signaling pathway is the main pathway of Ca^2+^ aggregation induced by PKC [9]: the activation of G protein requires the interaction of signal molecules on the outside of the membrane with the receptor of the G protein, and the activated G protein, in turn, activates the downstream phospholipase C (PLC). Phosphatidylinositol (PIP_2_) is decomposed by the activated phospholipase C, resulting in the formation of inositol triphosphate (IP_3_) that promotes the release of Ca^2+^ and diacylglycerol (DAG) that activates PKC. Ca^2+^ is released from the endoplasmic reticulum in large quantities and binds to calmodulin in the cytoplasm to participate in subsequent physiological and pathological processes. Under the coordination of Ca^2+^, the cascade of reaction caused by activated PKC reduces the permeability of cell membrane to calcium, promotes the massive influx of Ca^2+^ outside the membrane, aggravates calcium overload, and leads to the disorder of brain cell structure and functional metabolism. The above processes are shown in Figure 2.

### 3.2. Roles of PKC in CIRI

In addition to the above relationship, the following factors of CIRI are currently identified as being related to PKC.

#### 3.2.1. PKC and Neurotransmitters

Neurotransmitter release is regulated by PKC, and it, thus, acts on CIRI. In CIRI, cerebral ischemia leads to changes in cell bioelectricity, and the pathological slow wave is aggravated and sustained due to the secondary injury caused by reperfusion. Inhibitory amino acids (inhibitory neurotransmitters), such as alanine, glycine, taurine, and *γ*-aminobutyric acid, were significantly elevated in early CIRI [10]. The excitatory amino acids, such as glutamic acid and aspartic acid, decreased significantly after ischemia-reperfusion and more significantly with time [11]. Through the regulation of PKC, glutamate release increases during CIRI, while the reuptake by neuronal cells decreases, resulting in extracellular glutamate accumulation, neuronal toxicity, and brain tissue damage.

#### 3.2.2. PKC and Neuronal Receptors

PKC activates neuronal receptors, which accelerate brain damage [12]. Taking excitatory amino acids as an example, n-methyl-D-aspartate (NMDA) receptor, *α*-amino-3-hydroxymethyl-propionic acid (AMPA) receptor, and metabotropic receptor are three subtypes of excitatory amino acid receptors. The activation of these three subtypes is related to PKC regulation. For example, PKC can phosphorylate postsynaptic NMDA receptors, thereby activating them to excitatory neurotransmitters [13]. The AMPA receptor activated by PKC can enhance its affinity to glutamate, thus promoting the opening of Na^2+^ channels and the depolarization of neurons. At the same time, large amounts of water enter cells, causing acute swelling of neurons and accelerating brain damage.

#### 3.2.3. PKC and Protein Channels

Protein phosphorylation is an important method of protein activation [14], especially the activation of membrane receptors and channel proteins, and protein phosphorylation is inseparable from the regulation of PKC. Acid-sensing ion channels (ASICs), which sense the extracellular acidic environment, are highly expressed in the central nervous system [15], and their main functional subunit ASICs-*α* plays an important mediating role in the neuronal cell injury caused by cerebral ischemia. PKC regulates the activation of ASICs-*α* by phosphorylation of the NF-*κ*B signaling pathway molecules and promotes the expression of ASICs-*α*, thereby enhancing cell damage induced by cerebral ischemia [16].

### 3.3. Roles of PKC Isoenzymes in CIRI

Current studies suggest that multiple PKC isoenzymes may play roles in CIRI.

#### 3.3.1. PKC-*γ* in CIRI

PKC-*γ* exists only in the brain and spinal cord. It not only plays an important role in cell proliferation and differentiation but also mediates the release of neurotransmitters and the occurrence of tumors. PKC-*γ* is dependent on Ca^2+^ and sensitive to phorbol ester or DAG. The increase in intracellular calcium ions stimulates the translocation of PKC-*γ* to a certain extent, which is associated with the increase in intracellular PKC-*γ* expression after ischemia-reperfusion. As a protective response of cells against injury [17], the increase in the PKC-*γ* concentration increases the membrane content. If the PKC-*γ* gene is removed, its neuroprotective ability is weakened, making it vulnerable to ischemic injury, indicating that PKC-*γ* has a protective effect on neurons. This was confirmed in the mouse middle cerebral artery occlusion experiment conducted by Aronowski et al. [18].

#### 3.3.2. PKC-*δ* in CIRI

The activation of PKC-*δ* is independent of calcium ions, and PKC-*δ* widely exists in various tissues and cells. PKC-*δ* plays an important role in intracellular signal transduction pathways induced by various extracellular stimuli [19] and is an important mediator of CIRI. The activation of the NMDA receptor induces PKC-*δ* mRNA and protein expression during cerebral ischemia [12]. Studies found that PKC-*δ* induces CIRI by mediating an inflammatory response in PKC-*δ*-knockout mice. The transplantation of bone marrow from PKC-*δ*-knockout mice into wild mice alleviated ischemic injury [20], suggesting that PKC-*δ* promotes perfusion injury. Meanwhile, PKC-*δ* is also associated with apoptosis in the delayed phase of cerebral ischemia-reperfusion and is an important therapeutic target in stroke patients during the long time window.

#### 3.3.3. PKC-*ε* in CIRI

PKC-*ε* is abundant in endocrine, immune, and nerve cells and plays a role in cell proliferation and differentiation, gene expression, and certain other processes, covering a wide range. The activation of PKC-*ε* is also independent of calcium ions but sensitive to phorbol ester and DAG. A large number of studies have shown that PKC-*ε* is activated by adenosine through the signal transduction pathway [21] and regulates mitochondrial ATP-sensitive potassium channels, thus protecting neuronal cells from ischemic injury.

## 4. Structure and Function of HIF-1

Hypoxia-inducible factor-1 (HIF-1), which was first discovered by Semenza and Wang in 1992, can be detected in almost all human cells and is expressed stably in an anoxic environment. HIF-1 belongs to a class of heterologous protein dimers consisting of *α* and *β* subunits. The major common domains of HIF-1 are as follows [22]: ① the bHLH domain, or the N-amino terminal, can bind to the DNA; ② the PAS domain, or the intermediate region, is a key structural region for heterodimerization; and ③ C-carboxyl region, where proteins can bind to cofactors to regulate transcription.

**Figure 3:**
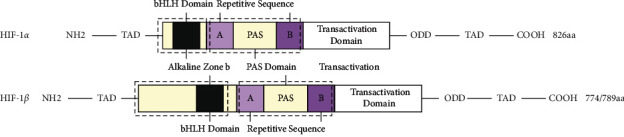
Schematic diagram of HIF-1 structure.

Of the two subunits of HIF-1, only the *α* subunit is functional, namely, HIF-1*α*. It senses the hypoxic signals at two terminal regions. An oxygen-dependent degradation (ODD) domain is located at the C-carboxyl region, which plays a variety of roles, including regulating transcription and stabilizing hypoxia-inducible proteins. Trans-activation domain (TAD) is present at both ends, and TAD-N plays a necessary role in activating transcription, while TAD-C has a fine regulation function. In terms of structure, HIF-1*β* is similar to the HIF-1*α* subunit, and its main function is to stabilize HIF-1 and promote conformational transition. In addition, the release of the *α* subunit is based on the binding of the *β* subunit to form a heterodimer. The structure of HIF-1 is shown in Figure 3.

It has been found that HIF-1*α* can effectively inhibit apoptosis under mild hypoxia. However, if cells are exposed to severe hypoxia for a long time, the overexpression of HIF-1*α* can induce apoptosis [23].

## 5. The Relationship between HIF-1 and CIRI

HIF-1 has been shown to regulate more than 100 downstream target genes, most of which are related to energy metabolism or oxygen supply, such as EPO, VEGF, GLUT, and apoptosis-related genes. When the body is hypoxic, HIF-1 specifically binds to the hypoxic-response elements contained in these genes. HIF-1 uses the protein products expressed by target genes to activate the body, including promoting the formation of blood vessels, upregulating the expression of red blood cells, increasing proliferation and differentiation of cells, regulating anaerobic metabolism, and decreasing apoptosis [24]. Thus, oxygen homeostasis in the hypoxic tissue can be maintained to counter the cascade of reactions caused by hypoxic ischemia.

By studying the animal model of focal CIRI, it was found that HIF-1*α* and VEGF levels were upregulated in the ischemic penumbra, which contributed to the formation of blood vessels to resist CIRI [25]. The survival of neurons in the ischemic brain tissue depends largely on the stability of HIF-1*α*. HIF-1*α* can effectively inhibit apoptosis, improve the survival rate of neuronal cells in mild hypoxia, and show a protective effect on the brain tissue [26].

Bcl-2 and Bax can regulate cell apoptosis; the former can inhibit cell apoptosis, but the latter promotes cell apoptosis. In the hypoxic state, the conformation of Bax changes and it enters the mitochondrial outer membrane, thus affecting mitochondrial permeability. At the same time, cytochrome C, generated by the mitochondria, is released from the transformation pore, enters the cytoplasm and binds caspase-9 and APAF-1 to form the apoptotic complex, activating the precursor caspase-9 [27]. Activated caspase-9 continues to activate the downstream precursor caspase-3, which can cleave several different protein substrates, resulting in apoptosis. Cyto C can not only activate caspase-3 but also produce a large number of free radicals via the peroxidation reaction, which blocks the generation of ATP in the mitochondria and impairs the function of mitochondria. Bcl-2 can bind Bax to form a heterodimer, which leads to the failure of mitochondrial permeability, thus inhibiting the generation of Cyto C in the mitochondria, weakening the caspase cascade amplification effect, and inhibiting apoptosis [28].

When brain tissue is under hypoxic environment, a large number of activated HIF-1*α* can upregulate the expression of Bcl-2 through the PI3K/Akt pathway, while the expression of Bax pro-apoptotic protein and NF-KB is downregulated, promoting the increase in the ratio of Bcl-2/Bax and effectively inhibiting the cascade amplification effect of caspase-3. At the same time, HIF-1*α* also effectively regulates caspase-3 activity via VEGF, TNF-*α* pathway, and ERK1/2 signaling pathway [29], thus weakening the cascade amplification effect of caspase and reducing nerve cell damage. In addition, HIF-1*α* also protects the damaged brain tissue by releasing antioxidants.

## 6. Association between PKC and HIF-1 in CIRI

Currently, there are only a few studies on PKC signal transduction and HIF-1 expression, especially on their association in CIRI. Certain studies targeting the most widespread intercellular protein, connexin 43 (Cx43), have shown that although Cx43 levels are significantly reduced in neurons in the damaged brain tissue, Cx40 levels are abnormally upregulated [30]. In addition, studies have found that the application of nonspecific gap junction blockers, such as 1-heptanol, can not only alleviate brain injury but also downregulate the expression of Cx43 [31], indicating that Cx43 may be one of the factors for the aggravation of brain tissue injury. Several studies have shown that HIF-1*α* can decrease Cx43 function, but the relationship between Cx43 phosphorylation and brain injury has not been clarified. Recent studies have shown that Cx43 initiates the abnormal remodeling of the Cx family of proteins in the central nervous system, so the abnormal expression of Cx proteins may be closely related to CIRI. Studies have also confirmed that Cx43 plays an important role in the maintenance of cell morphology, suggesting that Cx43 has an effective cell protection effect [31]. Elbadawy found that increased Cx43 phosphorylation specifically inhibited Cx43 function, thereby enhancing the expression of HIF-1*α* [32].

**Figure 4:**
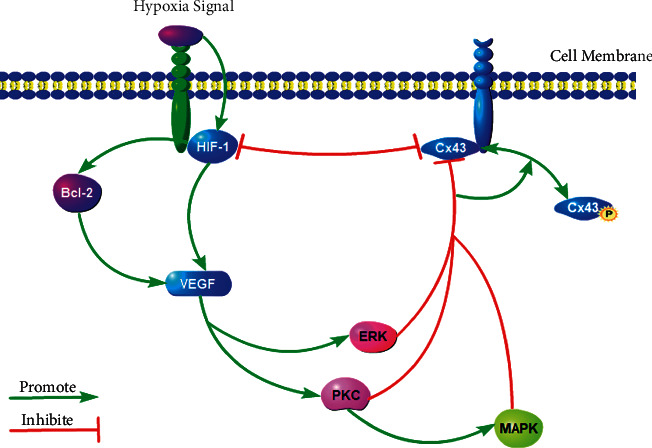
Association between PKC and HIF-1 in CIRI.

An increased level of activated HIF-1*α* upregulates VEGF expression [33], which activates the PKC and ERK pathways [34], leading to more extensive Cx43 phosphorylation. Ultimately, it worsens the dysfunction of brain tissues. The downregulation of HIF-1*α* expression in the hyperacute phase significantly reduced the phosphorylation of Cx43 protein and the expression of inflammatory factors in the hippocampus [35]. In conclusion, the downregulation of HIF-1*α* expression can inhibit Cx43 phosphorylation, thus inhibiting the secretion of inflammatory factors by the nerve cells. This regulatory effect plays a protective role against brain injury. It is also suggested that the PKC and HIF-1 signaling pathways have a certain association in CIRI. The association between PKC and HIF-1 in CIRI is shown in Figure 4.

## 7. The Effects of TCM Compounds on PKC and HIF-1

A few studies conducted on the treatment of CIRI caused by cerebral ischemia, stroke, cerebral embolism, and other diseases by TCM compounds found that the therapeutic effect of TCM compounds is remarkable. Among these medicinal compounds, a few TCM components mainly act on the PKC and HIF-1 signaling pathways to achieve the therapeutic effect on CIRI.

### 7.1. TCM Formulation Alleviates CIRI by Regulating the PKC Signaling Pathway

#### 7.1.1. Danhong Injection

Danhong injection is mainly extracted from *Salvia miltiorrhiza* and safflower and promotes blood circulation, alleviates blood stasis, and clears arteries [36]. It is widely used in the clinical treatment of cardiovascular and cerebrovascular diseases, especially in CIRI. PKC, an important signaling molecule in the development of CIRI, is essentially a multifunctional cytoplasmic enzyme. In the presence of a second messenger, PKC binds to the cell membrane, and cytoplasmic enzymes are activated to participate in various biochemical reactions and in the regulation of gene expression [37]. After the activation of PKC, cerebral vasoconstriction continues, resulting in CIRI [38]. Danhong injection significantly decreased the serum PKC content in patients. When Danhong injection was combined with butylphthalide, not only did the PKC content reduce significantly, but vasospasm was alleviated [39], suggesting that butylphthalide enhanced the efficacy of the Danhong injection.

Danhong injection is a commonly used compound preparation for the clinical treatment of CIRI since it inhibits the activity of PKC and the signaling pathway responsible for the occurrence and development of CIRI, to protect the brain cells. Butylphthalide can reduce the injury caused by cerebral ischemia by relieving the spasm and dilating the blood vessels. The efficacy and safety of Danhong injection were improved by combining butylphthalide, providing a novel drug using idea for the treatment of CIRI.

#### 7.1.2. Qi Leech Capsule

Qizhi capsule is composed of *Astragalus membranaceus*, leeches, *Salvia miltiorrhiza*, *Pheretima*, and ephedra, etc. and mainly plays the role of invigorating qi, alleviating blood stasis, activating blood circulation, and clearing collaterals, and is an effective drug for the treatment of cerebral ischemia [40]. The treatment of cerebral ischemia by Qizhi capsule mainly includes two aspects: one is to the inhibition of CIRI by tanshinone IIA in astragalus [41] and the other is regulating the activity of the Ca^2+^-PKC-MARCKS signaling pathway and the phosphorylation of related proteins [42]. Excessive Ca^2+^ activates PKC in large quantities, which in turn phosphorylates MARCKS proteins to produce a large number of P-MARCKS. Highly expressed P-MARCKS activates microglia in the ischemic areas [43], which damages the neurons and their function. In addition, another component of the Qizhi capsule, hirudin extracted from the leech, can also protect brain cells [44], reduce cerebral vascular thrombosis, increase brain blood supply, and alleviate ischemia-reperfusion injury.

Qizhi capsule can inhibit the phosphorylation of PKC, MARCKS, and other proteins and downregulate the overexpression of P-PKC and P-MARCKS proteins, thus blocking the PKC-MARCKS signaling pathway and alleviating CIRI. The components of the Qizhi capsule can effectively scavenge free radicals and reduce the damage due to lipid peroxidation to the vascular endothelium. It can also inhibit thrombin activity, reduce thrombosis, reduce vascular permeability, and protect the vascular endothelium and brain cells.

#### 7.1.3. Buyang Huanwu Decoction

Buyang Huanwu decoction is composed of *Astragalus membranaceus*, *Angelica sinensis*, *Ligusticum chuanxiong*, *Radix paeoniae*, peach kernel, safflower, and earthworm, which has the effects of invigorating qi, promoting blood circulation, and clearing collaterals. It is a famous Chinese herbal formula for treating CIRI. In CIRI, vascular endothelial cells (VECs) are severely damaged, but they play an important role in preventing the occurrence of nonadaptive coagulation [45]. The thrombin receptor can cleave phosphoinositol to produce IP_3_ and DAG. DAG activates PKC and other Ca^2+^-sensitive proteins. Therefore, it is an important pathway for thrombin to induce VEC activation through PKC. It was found that Buyang Huanwu decoction plays an antithrombotic role by inhibiting the activation of PKC induced by thrombin [46, 47], and the effective ingredients, alkaloids and glycosides, are responsible for the inhibiting effect.

Buyang Huanwu decoction can not only improve the activity of fibrinolytic enzyme but also inhibit platelet aggregation and enhance the activity of anticoagulants, making it effective for the treatment of deficiency of qi and blood stasis. The active ingredients of Buyang Huanwu decoction have a significant effect on the treatment of CIRI-induced thrombosis and endothelial cell injury. PKC plays an important role in thrombosis induced by thrombin, indicating that Buyang Huanwu decoction can downregulate the expression of PKC and inhibit the PKC signaling pathway activated by thrombin, thus playing an antithrombotic role.

#### 7.1.4. *Ixeris sonchifolia* Hance Injection

The *Ixeris sonchifolia Hance* injection is mainly extracted from *Ixeris sonchifolia Hance*, and has a complex chemical composition, including flavonoids, adenosine, triterpenoid saponins, and sesquiterpenoid lactones [48]. It removes heat and detoxifies the body, alleviates blood stasis, relieves pain, reduces swelling, helps in the drainage of pus, and is widely used to treat cardiovascular and cerebrovascular diseases [49]. The *Ixeris sonchifolia Hance* injection can inhibit the Rho A/ROK-*α* and PKC-*δ*/MARCKS signaling pathways in addition to scavenging free radicals, exerting antioxidant effects, and inhibiting calcium overload and inflammatory factors [50]. Among them, the Rho A/ROK-*α* signaling pathway is involved in various mechanisms in CIRI, such as vasospasm [51], thrombosis [52], oxidative stress [53], and the promotion of apoptosis [54], while the PKC-*δ*/MARCKS signaling pathway also promotes CIRI. A study found that there was an interaction between the two pathways [55] and ROK-*α* can promote the activation of PKC-*δ*, which phosphorylates MARCKS and damages the brain cells. The *Ixeris sonchifolia Hance* injection can reduce the expression of the ROK-*α* protein and mRNA, inhibit the phosphorylation of PKC-*δ* and MARCKS, and play a protective role in cerebral ischemia. Studies have found that the PKC/MARCKS signaling pathway can induce hypoxia injury in cerebrovascular endothelial cells, but the *Ixeris sonchifolia Hance* injection can reduce the expression of P-PKC and P-MARCKS to inhibit the PKC/MARCKS signaling pathway and protect the vascular endothelial cells [56, 57].

The protective effect of the *Ixeris sonchifolia Hance* injection on hypoxic cells is bifold. On the one hand, the ROK-*α* expression was decreased by downregulating the activities of the Rho A/ROK-*α* and PKC-*δ*/MARCKS signal pathways, and the phosphorylation of PKC-*δ* and MARCKS was decreased by the interaction, thus alleviating cerebral ischemia cell damage. On the other hand, it inhibits the PKC/MARCKS signaling pathway directly to protect the hypoxic cells.

### 7.2. TCM Formulation Alleviates CIRI by Regulating the HIF-1 Signaling Pathway

#### 7.2.1. Compound Danshen Tablet and Danhong Injection

The main ingredient of the compound Danshen tablet is *Salvia miltiorrhiza*, which has demonstrated a beneficial clinical effect on CIRI and promotes blood circulation and removes stasis, regulating qi and relieving pain. The active component of Danshen is mainly tanshinone, which can promote the high expression of HIF-*α* during cerebral ischemia [58, 59] and the generation of blood vessels in ischemic tissues, and improve microcirculation through the HIF-1/VEGF signaling pathway [60]. At the same time, the transcription of a series of genes downstream of HIF-1 is activated to increase the secretion of erythropoietin [61], induce angiogenesis, repair the damaged vascular endothelium, etc. In addition, the expression of GLUT1 and glycolytic enzymes also increases [62], to exert the antioxidant effect after reperfusion and improve brain tissue energy metabolism to increase oxygen supply to the brain tissue, improve microcirculation, and reduce brain damage. The main components of Danhong injection, Danshen and safflower, can activate the PI3K/AKT signaling pathway [36], upregulate the activities of P-AKT and HIF-1*α* proteins, decrease the expression of caspase-3 [63], reduce the apoptosis of nerve cells, and protect brain tissue.

In the ischemic brain tissue, both compound Danshen tablet and Danhong injection can act on the HIF-1 pathway to maintain the normal function of brain tissue by promoting increased expression of HIF-1*α* to initiate the expression of downstream genes, thus increasing the generation of collateral circulation vessels to improve the hypoxia tolerance of nerve cells and reduce apoptosis.

#### 7.2.2. Longzhi Decoction

Longzhi decoction inherited the function of Buyang Huanwu decoction to promote blood circulation and dissipate blood stasis, and the increased herbs, cyathula root, and leeches further enhanced the function of alleviating blood stasis and facilitating menstruation [64], making it an effective Chinese medicine preparation for the treatment of acute stroke. A study found that the treatment of Longzhi decoction can significantly improve the level of HIF-1 [65] and the content of downstream VEGF [66]. VEGF is a downstream gene activated by HIF-1*α*. It can promote microvascular formation through FIk-1 receptor protein [67, 68], improve microcirculation of ischemic focus, and accelerate the recovery of neurological function in the cerebral ischemic tissue. As another representative angiogenic factor, Ang-2, which is specifically expressed by the endothelial cells, is significantly expressed in the ischemic brain tissue after treatment with Longzhi decoction. A large amount of Ang-2 can promote angiogenesis and nerve repair [69, 70], eliminate the effect of VEGF on vascular permeability of diseased tissues [71], and maintain the stability of neovascularization.

Longzhi decoction can affect the expression of VEGF and its receptor genes in tissues around the cerebral infarct area through the HIF-1*α* signaling pathway. The increased expression of HIF-1*α* promotes the expression of VEGF, which is beneficial to the formation of ischemic focal vessels. Longzhi decoction can also improve the expression of ANG-2, synergistic with HIF-1*α* and VEGF, to promote angiogenesis in ischemic and its surrounding areas, protect vascular units, reduce the increase in vascular permeability induced by VEGF, and play a neuroprotective role.

#### 7.2.3. Yangyin Tongnao Granule

Yangyin Tongnao granule is a TCM compound preparation mainly composed of *Rehmannia glutinosa*, *Astragalus membranaceus*, *Ligusticum chuanxiong*, and Pueraria root [72]. The main effective components in the compound are catalpol, astragaloside IV, ligustrazine, puerarin, and ferulic acid. The treatment effect of Yangyin Tongnao granule is demonstrated, and it shows the effects of dilating blood vessels, inhibiting thrombosis, and improving microcirculation and neuroprotection [73]. Experimental studies have shown that serum HIF-1*α* and VEGF levels in acute cerebral ischemia-reperfusion rats can be significantly increased by the treatment of Yangyin Tongnao granule [74–76], indicating that the active components of Yangyin Tongnao granule have a good therapeutic effect in brain injury repair. Inflammation is closely involved in the occurrence and development of ischemia-reperfusion injury [72, 77]. The intracytoplasmic pattern recognition receptor, NOD-like receptor (NLRP3), is activated when cells are infected and bind to pro-caspase-1 to form the NLRP3 inflammasome. NLRP3 inflammasome activates pro-caspase-1 to form caspase-1, which decomposes inflammatory mediators, such as IL-1*β* and IL-18 precursors, into inflammatory factors with important biological activity [78], participating in various inflammatory processes and aggravating CIRI. The treatment with Yangyin Tongnao granule can significantly reduce the content of NLRP3 inflammasome [79], and a large number of activated HIF-1*α* can inhibit the formation of caspase-1 through the PI3K/Akt/Bcl-2 pathway [28], blocking the occurrence of the above cascade process, thus playing a protective role in the ischemic brain tissue.

Yangyin Tongnao granules can increase the expression of HIF-1*α* and VEGF in ischemic lesions, promote the formation of new blood vessels, relieve ischemia and hypoxia, and improve microcirculation due to the combined action of the main active ingredients of different TCMs. At the same time, it inhibits the activation of the NLRP3 inflammasome, reduces the formation of caspase-1 via different signal pathways, and decreases the inflammatory response of CIRI, which is conducive to the repair and functional recovery in the nerve tissue of the ischemic focus.

#### 7.2.4. Ginsenoside

Ginsenosides are the main biologically active components of ginseng [80], which are categorized into Rg1-3, Rb1-2, and Rc types. These triterpene saponins have been shown to have many beneficial effects on the human body, especially on the nervous system. Ginsenosides can reduce excitotoxicity, oxidative stress, and neuroinflammation, maintain neurotransmitter balance, resist apoptosis, and maintain mitochondrial stability [81], which contribute to effectively reducing CIRI and protecting the nervous system [82, 83]. Studies have shown that ginsenosides induce the large amounts of expression of HIF-1*α* in large amounts through the HIF-1*α*/VEGF signaling pathway [84, 85]. As a downstream target gene of HIF-1, VEGF can be transcriptionally activated to enhance angiogenesis and reduce CIRI. At the same time, the high expression of HIF-1*α* and VEGF induced by ginsenosides can increase the proliferation and differentiation of neural stem cells in ischemic and hypoxic brain tissues [86, 87], promote the repair and generation of nerves, and help the recovery of brain function.

Ginsenoside regulates the transcription of the HIF-1*α* gene and initiates the transcription of the downstream target gene VEGF, which can not only promote collateral circulation via the formation of new blood vessels but also improve the ischemic and hypoxic state of microcirculation. At the same time, it acts on neural stem cells to promote their proliferation and differentiation to repair brain damage and restore brain function.

### 7.3. TCM Formulation Alleviates CIRI by Regulating the PKC/HIF-1 Signaling Pathway

Guhong injection (GHI) is a compound preparation composed of safflower extract (a TCM) and acetylglutamine (Western medicine) [88]. It has anti-inflammatory and antioxidant effects; therefore, it is widely used in the treatment of cerebrovascular diseases [89]. Hypoxia is a key factor in the survival of cells injured by ischemia. Studies have shown that GHI can reduce the concentration of HIF-1*α*, PKC, and EPO in the serum and inhibit the expression of HIF-1*α* mRNA and protein in the brain [90]. These results indicated that GHI has a protective effect on CIRI by inhibiting the PKC/HIF-1*α* signaling pathway. Nicotinamide adenine dinucleotide phosphate oxidase 4 (NOX4) is the main oxidative enzyme in the pathogenesis of cerebral ischemia [91]. PKC plays a major role in the NADPH oxidase signaling pathway, and in the MAPK and NF-KB signaling pathways [92]. In prolonged cerebral ischemia, the level of prolyl hydroxylase 2 (PHD2) decreases, probably because PHD2 is the transcription target of HIF-1*α* [90]. When the expression of HIF-1*α* decreases, PHD2 also decreases. PKC inhibits the activation of PHD2, while HIF-1*α* induces the hydroxylation and degradation of PHD2 [93]. HIF-1*α* protects gene transcription by regulating the adaptive response to hypoxia and other stresses and plays a key role in the activation of endogenous substances downstream of cerebral ischemia. Studies have shown that EPO and iNOS are the key downstream substances activated by HIF-1*α* [94]. In addition, EPO is an inducer of ischemic tolerance and can prevent the death of apoptotic cells. Therefore, GHI upregulates the expression of EPO and iNOS in the brain of rats with cerebral ischemia injury.

GHI induces a protective effect on cerebral ischemia via the PKC/HIF-1*α* signaling pathway, which may be a potential mechanism for GHI to alleviate CIRI. Therefore, in addition to treating ischemic stroke, GHI also has the potential to treat cerebrovascular diseases. As a combination of Chinese and Western medicine, GHI has more multitarget pharmacological effects. Therefore, other mechanisms underlying the protective effect of GHI on cerebral ischemia are worthy of further study.

## 8. Problems and Prospects

PKC and HIF-1 are highly expressed in cerebral ischemia and reperfusion, and there is a special relationship between the two signaling pathways and comprising factors. The interaction and mechanism between the two are of great importance to elucidate CIRI. The elucidation of the relationship between PKC and HIF-1 is expected to identify novel therapeutic targets, which is of great significance to the prognosis and treatment of CIRI. Based on the result of treatment of CIRI using TCM compounds targeting PKC and HIF-1, research on the new TCM compounds is needed to improve the therapeutic effect on CIRI.

Western medicines usually work via a single therapeutic target that is influenced by the patient's genotype and comorbidities. Because the metabolic rate of drugs varies greatly among different populations, there is the individualization of drugs. The treatment of TCM is usually by different compatibility of TCM compound. Compared to single-target Western medicine, TCM compound, which is a multicomponent and multitarget drug preparation, may have a more extensive clinical application.

As an important supplement or substitute for Western medicine, TCM preparations are widely used in clinical practice (mainly in China) to improve the clinical outcome of patients with cerebrovascular diseases. These protective mechanisms include the inhibition of oxidative stress and inflammation, anticoagulant and antithrombotic effects, reduction in apoptosis and blood pressure, dilation of blood vessels, and promotion of angiogenesis. Since most clinical trials are conducted in China and the sample size is limited, the efficacy and safety of TCM compounds still need to be further verified through high-quality, large-scale, multicenter, and randomized controlled clinical trials. In addition, due to the multicomponent nature of TCM compounds, the multitarget mechanism in cerebrovascular diseases is still not clear. Further molecular, cellular, and animal studies are needed to elucidate their multipharmacological characteristics and accelerate the overall development and utilization of TCM preparations.

The study of the combination of TCM and other Western medicine is an important direction, which may enhance the therapeutic effect of drug preparation. The emergence of systematic and network pharmacology will also provide new insights into the therapeutic potential of TCM preparations in cerebrovascular diseases. Therefore, the development and utilization of TCM compounds in cerebrovascular therapy have laid a good foundation for the development of TCM.

## Data Availability

No data were used to support this study.
